# Influencing factors of and driving strategies for vegetable farmers' green pesticide application behavior

**DOI:** 10.3389/fpubh.2022.907788

**Published:** 2022-09-08

**Authors:** Yun Teng, Xinlin Chen, Yue Jin, Zhigang Yu, Xiangyu Guo

**Affiliations:** ^1^College of Engineering, Northeast Agricultural University, Harbin, China; ^2^Postdoctoral Mobile Station of Agricultural and Forestry Economic Management, Northeast Agricultural University, Harbin, China; ^3^College of Economics and Management, Northeast Agricultural University, Harbin, China

**Keywords:** vegetable safety, agricultural product quality and safety, farmer behavior, driving strategy, structural equation

## Abstract

At present, the phenomenon of excessive pesticide residues in vegetables is prominent, causing widespread concern among all sectors of society. Excavate the influencing factors in the farmers themselves, government, market and society that affect vegetable farmers' green pesticide application behavior, clarify the influence mechanism of influencing factors on vegetable farmers' green pesticide application behavior. The study includes two parts: First, Grounded theory is used to construct a conceptual model that illustrates vegetable farmers' green pesticide application behavior. The second part applies the structural equation modeling to verify the research hypotheses, and reveals various factors in vegetable farmers' green pesticide application behavior (GB). The Results: Behavioral attitude (BA) and behavioral feedback perception (BP) are precursor variables that determine vegetable farmers' green pesticide application motivation (GM), and thus affect vegetable farmers' GB. Government supervision and regulation (GR), and market adjustment guidance (MG) are external factors that regulate the strength of the relationship between GM and GB. It is necessary to further strengthen the reference and normative role of society in vegetable farmers' GB, and provide a driving strategy for vegetable farmers' GB. Thus, it can better improve the quality of pesticide application and ensure vegetable safety.

## Introduction

At the present stage, China's economic and social development is in a complex environment of multi-center interdependence (1). The continuous increase in agricultural product demand has formed an inertial dependence on the use of pesticides and other chemicals (2). China has 7% of the world's arable land, however China's use of chemical fertilizers and pesticides amounts to 35% of the global total. The direct and indirect pollution caused by excessive pesticide residues of agricultural workers forms a vicious circle, attracting great attention and arousing concern from all sectors of society (3–5). Agricultural production is dominated by large quantities and small scales, and the characteristics of “farmer dispersion and discrete markets” are prominent. Thus, the government cannot fully monitor and control vegetable farmers' pesticide application behavior (6, 7). Excessive pesticide residues affect environmental pollution, vegetable safety and human health (8). In China, the types of pesticides application are complex and diverse. Pesticide types include procymidon, carbendazim, metalaxyl, difenoconazole, alachlor, propamocarb, dimethomorph, Pyraclostrobin, propiconazole and pyridaben, etc. Green pesticide application behavior refers to the application of these pesticides on time and at the proper dose, which can not only produce high targeted insecticidal and herbicidal effects, but also reduce their toxic side effects. Retail vegetable farmers constitute the main group using pesticides. Vegetable farmers often ignore the quality and safety of agricultural products in the production process, because they are driven by profit. They also tend to use highly toxic and violently toxic pesticides to seek economic income that is stable and as high as possible. This behavior has a decisive influence on the formation of pesticide residues, and the standard is even exceeded (9). We have investigated the pesticide residues in several vegetables, and the results are shown in Table 1. From Table 1, we can find that more than half of the pesticide residues in vegetables are not up to standard, and the problem of pesticide residues in vegetables is serious, which needs to be solved. At present, in China's agricultural development, the quality orientation of agricultural products is the most important issue, and reducing pesticide application is a key way to alleviate the serious vegetable safety problems and ecological environment deterioration risks facing agricultural development. To ensure the quality and safety of agricultural products, strictly controlling farmers' pesticide application has become the top priority (1, 4).

**Table 1:** Types and residues of pesticides detected in vegetables.

**Pesticide category**	**Vegetables**	**Pesticide test results (mg/kg)**	**Pesticide limit requirements (mg/kg)**	**Result**
Procymidon	Eggplant	0.19	0.14	Qualified
Carbendazim	Tomato	0.20	0.26	Unqualified
Metalaxyl	Cucumber	0.23	0.45	Unqualified
Difenoconazole	Chili	0.10	0.03	Qualified
Propamocarb	Broccoli	0.25	0.31	Unqualified
Dimethomorph	Romaine lettuce	0.35	0.42	Unqualified

In this research, vegetable farmers' green pesticide application behavior needs to the goal of “low residue, low pollution, ensuring quality, and ensuring safety.” This goal includes taking the initiative to select approved pesticides, reducing pesticide application volume and frequency, applying pesticides at safe intervals, avoiding vegetable pesticide residues that exceed the standard, and ensuring the quality and safety of vegetable production activities. Accordingly, this study explores the internal and external factors in vegetable farmers' GB from a micro level, and it explores the influencing factors of vegetable farmers' GB through in-depth interviews. Based on the systematic collection of interview records, grounded theory is used to refine the factors of vegetable farmers' GB. Additionally, the structural equation modeling (SEM) is used for empirical verification to provide a scientific management and decision-making basis for constructing a driving strategy for green pesticide application for vegetable farmers.

## Literature review

In Silent Spring, Carson described the enormous damage to the human body and ecological environment caused by highly toxic pesticides. Since then, the harm caused by unreasonable pesticide application has gradually become a concern of scholars in China and in other countries (10), including scholars in economics, management, marketing, psychology, praxeology and other disciplines (11). Research on the government supervision system for agricultural worker has been conducted at macro disagreement (12), while research on effective methods of encouraging agricultural worker to standardize the application of pesticides and fertilizers has been conducted at the micro level. From the micro level, the research on agricultural workers' pesticide application behavior is mainly based on the school of organizational production represented by Chayanov, the rational small farming school represented by Schultz, and the classic farmer behavior theory of the historical school represented by Huang Zongzhi, The first school believes that the behavior of agricultural worker is not to pursue the maximization of market profits, but to pursue the balance between consumption satisfaction and labor hardship; The second school believes that behavior of agricultural worker is completely rational and makes behavioral decisions with the goal of pursuing maximum profit; The third school puts forward a relatively Commodity Smallholder Theory, and believes that agricultural worker are both profit-seeking and livelihood producers, their decision behavior is affected by both economic and non-economic factors (13). In addition, based on behavior motivation theory, the theory of planned behavior theory, producer behavior theory, game theory, and information asymmetry theory, scholars have focused on the purpose, difference, reality and economics of vegetable farmers' pesticide application behavior, but they have not reached a unified conclusion (14). A consensus has been reached with regard to the notion that the pesticide application behavior of vegetable farmers is a key factor in vegetable quality and safety. Therefore, controlling and regulating vegetable farmers' pesticide application behavior are an important condition of vegetable quality and safety (15).

This study explores the factors in vegetable farmers' pesticide application behavior. The influencing factors of farmers' pesticide application decisions include economic factors, social factors and the internal factors (12). The characteristics of farmers, such as their gender, age, farming experience, family characteristics and educational level, affect their safe pesticide application behavior (16). Risk perception, safe pesticide application awareness and safe pesticide application motivation have important effects on farmers' pesticide application behavior (17). The influencing factors of farmers' pesticide application behavior involve information sharing, information symmetry, agricultural insurance, pesticide residue detection and other factors (18). Farmers' pesticide application behavior is closely related to governmental attention (19). By considering the farmers application behavior, the government could have a more positive impact by strengthening legislation on and establish institutions for the quality and safety of agricultural products, and strictly supervising the behavior of agricultural producers (20, 21). The government can reduce the costs of supervision, increase agricultural subsidies, realize complete information sharing, increase farmers' additional income, reduce the costs of production inputs and take other measures to raise farmers' green pesticide application enthusiasm (22). Having a sound sales channel for high-quality agricultural products, supporting quality control standards and agricultural product quality traceability systems, and increasing consumer' purchases and willingness to pay are favorable conditions that will drive farmers to standardize their pesticide application (23). Under a situation of information asymmetry, it is difficult for consumers to distinguish the quality of agricultural products. Establishing market access systems and agricultural product quality identification systems can encourage farmers to produce high-quality agricultural products (24, 25).

## Methodology

### Research method

**Figure 1:**
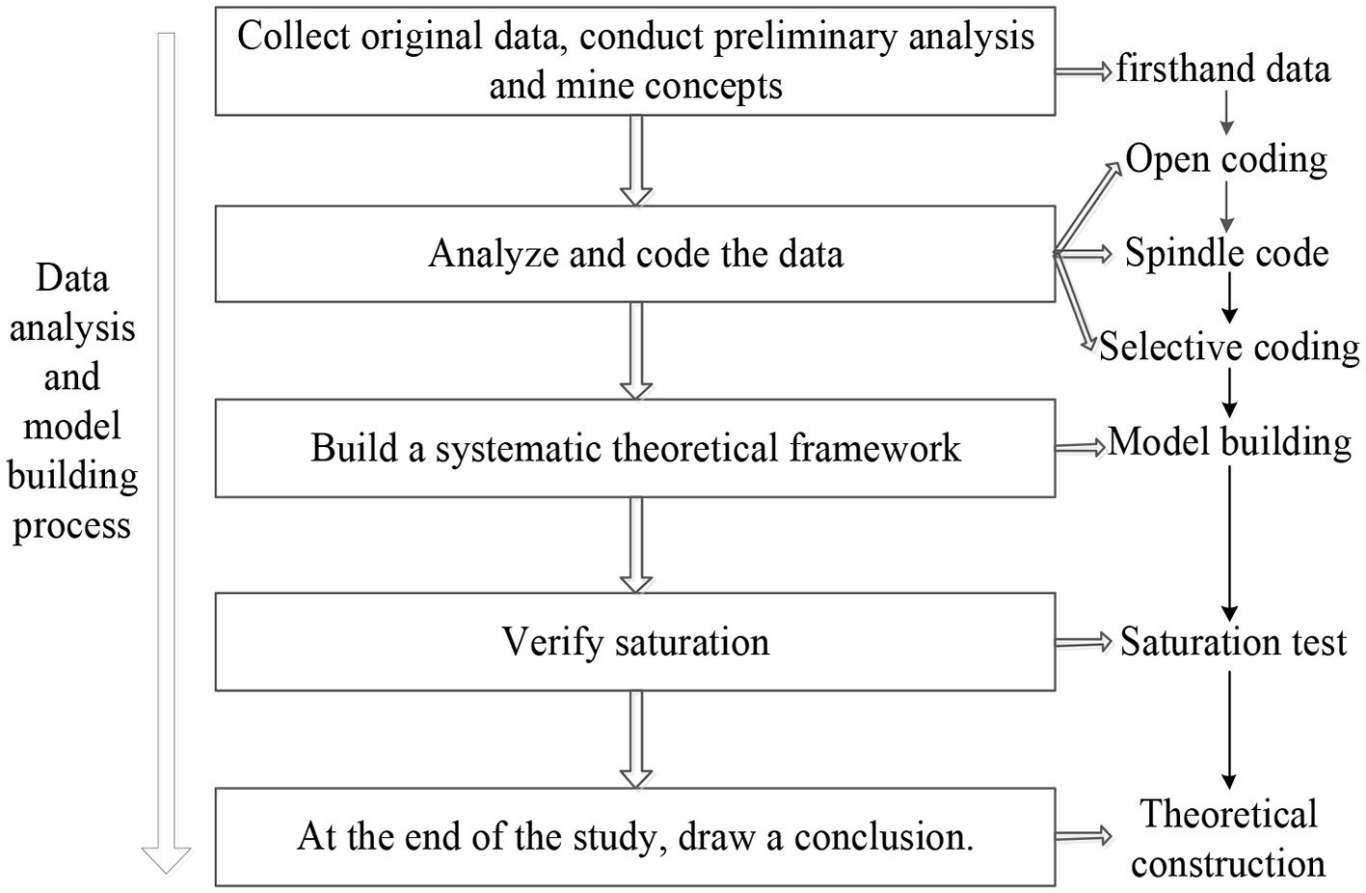
The coding process of grounded theory.

Grounded theory, which is used to qualitatively analyze the information collected by researchers, was proposed by sociologist Barney Glaser et al. This method is characterized by strong theoretical tension and a strong ability to deduce exploratory theory (26). Grounded theory is a way of qualitative research. Its main purpose is to establish a theory based on empirical data. Researchers generally do not have theoretical assumptions before the start of research. They start with actual observation, summarize experience from the original data, and then rise to the systematic theory. It constructs a theoretical system through three links: open coding, spindle coding, and selective coding. In the process of data analysis, continuous comparative analysis is adopted until a new substantial theoretical framework is constructed (27). The specific process of Grounded theory is shown in Figure 1. In this study, the data collection process mainly involved three methods: face-to-face interviews, voice interviews and focus group interviews. The face-to-face interviews mainly consisted of meeting with farmers, effectively adjusting the content of the interviews in real time, and observing non-verbal behaviors. Each interview lasted approximately 30 min. The voice interviews involved using WeChat voice technology, and the interviewees were more willing to express their inner thoughts. Each interview lasted approximately 30 min. The focus group interviews were conducted in two groups of 5 people under the guidance, and each group met for 1 h. The face-to-face interviews, voice interviews and focus group interviews were adopted to comprehensively and systematically collect interview records, and to establish a theoretical model.

In the second part of this study, SEM was used to quantitatively research the conceptual model derived from the grounded theory. Structural equation model (SEM) is a statistical analysis method to identify, estimate and verify the complex theoretical hypothesis model. Structural equation methods is based on questionnaire survey, combined with regression analysis, factor analysis and path analysis to verify the complex theoretical hypothesis model, and can verify the influence relationship and influence effect between variables based on a large number of data. Based on the grounded theory in the previous step, prepare a questionnaire to obtain interview records and draw research hypotheses. Since the questionnaire items need to be related to the categories of the conceptual framework, the key issues of each category were highlighted and designed through reverse transcription. The questionnaire analysis consisted of two parts: the first part consisted of basic data statistics, the second part consisted of hypothesis testing. In the survey, the interviewees were asked to use a 5-point Likert scale anchored by 1 “strongly disagree” and 5 “strongly agree” to rate their level of agreement with particular items. A neutral item “uncertain” was included to prevent the respondents from providing biased results due to exact choices. Before starting the SEM analysis, IBM SPSS 22 was used to analyze the data. The SEM and IBM SPSS 22 were used to verify the causal relationships and pathways between the variables in the conceptual model.

### Sample

In the qualitative phase of this research, the first part consisted of selecting interview samples. We selected Shandong, Hebei, Henan and Heilongjiang Province as research samples. Shandong, Hebei and Henan Province are the main vegetable production areas in China. Although the vegetable production of Heilongjiang Province ranks in the middle among Chinese provinces, the vegetable industry is one of the leading industries in the agriculture of Heilongjiang Province and plays a decisive role in increasing farmers' income. During the survey process, all the vegetable farmers interviewed had experience with pesticides application. The survey adopted a combination of stratified random sampling and random sampling to select samples, and the prefecture-level municipal administrative regions under the jurisdiction of each province were selected as sample cities (districts). China has a large area and a large number of farmers, so it is impossible to investigate all the farmers. Meanwhile, China's administrative regions are divided into five levels: province, city (districts), county, town and village. Stratified sampling is used to sample from each administrative region, and then random sampling is carried out. Stratified random sampling and random sampling are conform to the requirements of wide, system and rationality of the research sample. The administrative regions of prefecture-level cities under the jurisdiction of each province are selected as the sample cities (districts). Two townships were randomly selected from each city (district), one administrative village was randomly selected from each town, and several eligible farmers were randomly selected from each village. A total of 125 vegetable farmers were selected.

In the quantitative phase of this research, the second part consisted of the questionnaire item selection. The questionnaire was designed based on the research hypotheses drawn in the first part, and also adopted a combination of stratified random sampling and random sampling to conduct large-scale questionnaire surveys with vegetable farmers in Shandong, Hebei, Henan and Heilongjiang provinces. Data collection used the 5-point Likert scale, and took field research and collected the questionnaire on site. A total of 700 questionnaires were distributed, and 698 were recovered. Due to the limitation of some farmers knowledge level and to fill in the questionnaire of emotional factors, follow the screening criteria of invalid questionnaire to eliminate information incomplete and inconsistent questionnaire, delete the data missing and incomplete questionnaire answered clearly is not consistent with actual situation of extreme questionnaire, 38 questionnaires finally selected is invalid, leaving 660 questionnaires for analysis, resulting in an effective response rate of 94.3%. The information of the sample is shown in Table 2.

**Table 2:** Demographics of the sample (*n* = 660).

**Characteristic**	**% of sample**	**Characteristic**	**% of sample**	**Characteristic**	**% of sample**	**Characteristic**	**% of sample**
Gender		Age		Planting years		Educational level	
Female	51.6	18–30 years	8.3	1–5 years	20.6	Less than primary school education	14.9
Male	48.4	31–40 years	19.7	6–10 years	30.5	Primary school education	48.6
		41–50 years	37.6	11–15 years	29.2	Secondary school education	31.7
		51–60 years	34.4	Over 15 years	19.7	University education	4.8
						Graduate-level education	0.0

## Results

### Conceptual model construction

The first phase of analysis consisted of open coding, which is the process of conceptualizing and categorizing original interview material. A total of 1,031 initial concepts were abstracted, and they were further categorized through cluster analysis to form 28 categories. To save space, three original statements and their initial concepts were selected for each category.

In the second phase, on the basis of open coding, spindle coding discriminates the correlations and relations among the independent categories, and excavates and establishes the potential logical relationships among the categories. Through analysis, it is found that each of the different categories obtained in open coding has an internal connection at the conceptual level. These internal connections were further aggregated and classified, resulting in 5 main categories. The meaning of each category and its corresponding open coding category are shown in Table 3.

**Table 3:** Main categories formed through spindle coding.

**Category**	**Main category**	**Initial category**
Internal factors	Behavioral attitude (BA)	Pesticide residues awareness (PRA) Pest and disease awareness (PDA) Environmental pollution awareness (EPA) Health hazard awareness (HHA) Social responsibility awareness (SRA) Knowledge ability level (KAL)
	Behavioral feedback perception (BP)	Behavioral risk perception (BRP) Behavioral loss and profit Perception (BLPP) Behavioral solidarity perception (BSP) Behavioral disturbance perception (BDP)
External factor	Government supervision and Regulation (GR)	Legal and regulatory enforcement (LRE) Regulatory penalty constraints (RPC) Incentive subsidy support (ISS) Pesticide operation standardization (POS) Quality traceability system (QTS) Publicity technology promotion (PTP)
	Market adjustment guidance (MG)	Quality control entry market (QCEM) Quality mark identification (QMI) Price based on quality (PBQ) Key risk control (KRC) Sales transaction model (STM) Consumer acceptance degree (CAD)
	Social reference specification (SS)	Media communication guide (MCG) Mass supervision report (MSR) Small farmer culture (SFC) Social moral constraints (SMC) Herd shortcut effect (HSE)

In the third phase, selective coding, which is based on spindle coding, explores the relationships between categories and excavates the core categories from the main categories. We analyzed the relationships between the main categories and core categories, and described the whole logical relationship and context in the form of storylines. A storyline is the construction of a new substantive theoretical framework. The logical context in this research storyline reflects the influencing factors and pathways of vegetable farmers' green pesticide application behavior (GB), as shown in Table 4.

**Table 4:** Storylines (typical logical relationships).

**Typical logical relationships**	**Meaning of the structural relationship**
Behavioral attitude→ Green pesticide application motivation Behavioral feedback perception→ green pesticide application motivation	Behavioral attitude and behavioral feedback perception are the precursor variables of vegetable farmers' green pesticide application motivation, directly determining the strength of their green pesticide application motivation.
Green pesticide application motivation→ Green pesticide application behavior	Vegetable farmers' green pesticide application motivation is a direct determinant of green pesticide application behavior, determining the emergence of green pesticide application behavior.
Government supervision and regulation ↓ Green pesticide application motivation→ green pesticide application behavior	Government supervision and regulation are an external context variable for vegetable farmers' green pesticide application behavior. It can regulate the relationship between vegetable farmers' green pesticide application motivation and behavior.
Market adjustment guidance ↓ Green pesticide application motivation→ green pesticide application behavior	Market adjustment guidance is an external context variable for vegetable farmers' green application behavior. It can regulate the relationship between vegetable farmers' green pesticide application motivation and behavior.
Social reference specification ↓ Green pesticide application motivation→ green pesticide application behavior	Social reference specification is an external context variable for vegetable farmers' green application behavior. It can regulate the relationship between vegetable farmers' green pesticide application motivation and behavior.

In the fourth stage, we used the remaining 1/4 of the interview records for test the theoretical saturation. The results showed that no new concepts were found, and no new logical relationships appeared in the existing main categories, indicating that the attribution model of vegetable farmers' green pesticide application behavior has good theoretical saturation.

**Figure 2:**
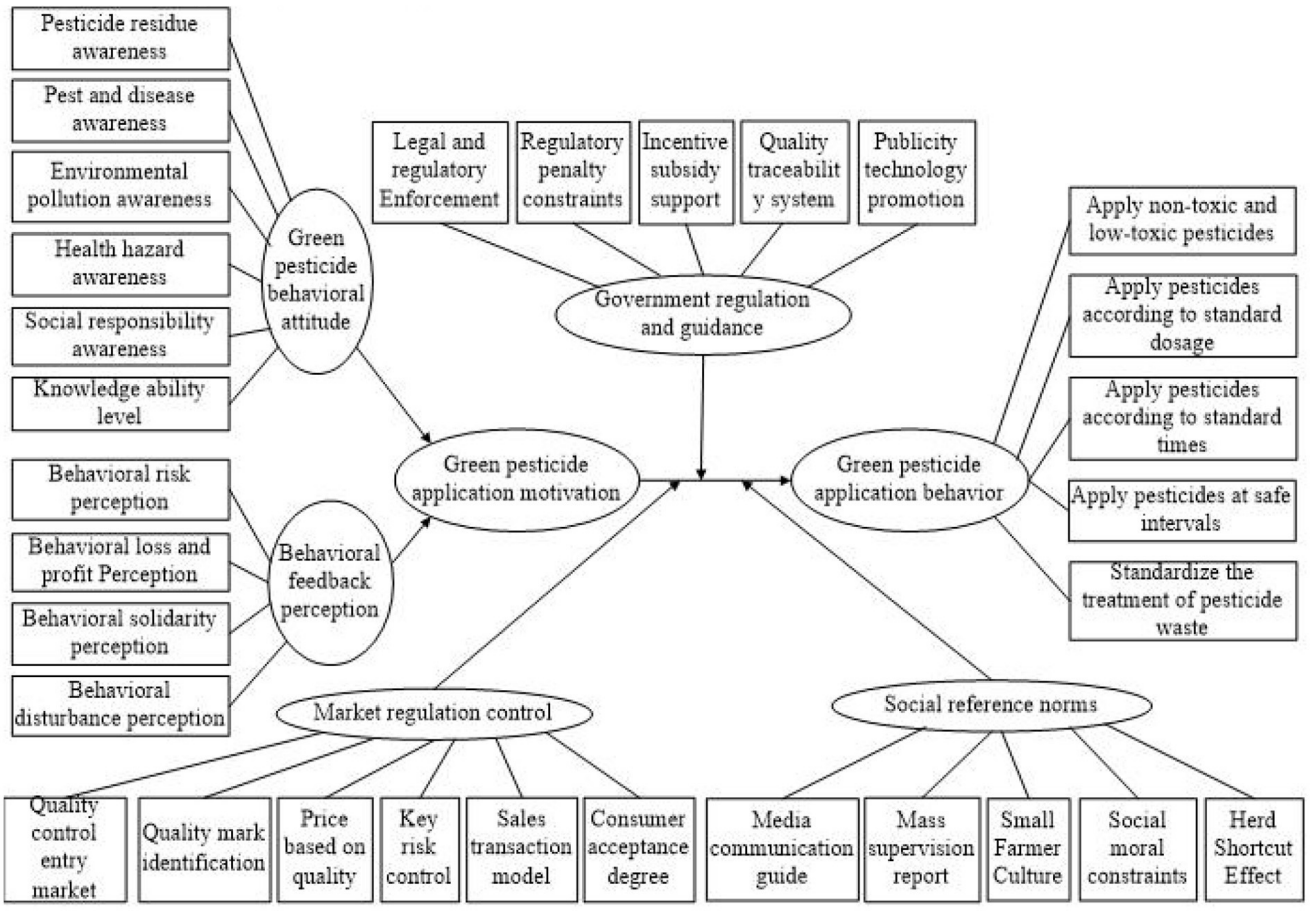
Conceptual framework.

Seven categories were generated at the end of the data analysis, behavioral attitude (BA), behavioral feedback perception (BP), government supervision and regulation (GR), market adjustment guidance (MG), social reference specification (SS), green pesticide application motivation (GM), and green pesticide application behavior (GB). The categories were arranged in graphical order based on the associations between them. Through the typical logical relationships in the storylines, the core category of the “attribution model construction and driving strategy of vegetable farmers” green application behaviors can be determined, and the psychological attribution and driving path of vegetable farmers' green application behaviors can be formed, which can be further summarized as the “(environment, needs) -motivation-behavior” chain. The conceptual model is shown in Figure 2. Based on the results of the grounded theory, the following hypotheses are proposed:

H1: Behavioral attitude has positive effect on green pesticide application behavior.

H2: Behavioral attitude has positive effect on green pesticide application motivation.

H3: Behavioral feedback perception has negative effect on green pesticide application behavior.

H4: Behavioral feedback perception has negative effect on green pesticide application motivation.

H5: Green pesticide application motivation has positive effect on green pesticide application behavior.

H6: Green pesticide application motivation plays an intermediary role between behavioral attitude and green pesticide application behavior.

H7: Green pesticide application motivation plays an intermediary role between behavioral feedback perception and green pesticide application behavior.

H8: Government supervision and regulation plays a regulatory role between green pesticide application motivation and green pesticide application behavior.

H9: Market adjustment guidance plays a regulatory role between green pesticide application motivation and green pesticide application behavior.

H10: Social reference specification plays a regulatory role between green pesticide application motivation and green pesticide application behavior.

### Empirical analysis

#### Reliability and validity test

SPSS24.0 software was used to test the reliability and validity of the questionnaire, and the specific data are shown in Tables 5, 6. Reliability testing uses Cronbach's alpha as an indicator, and the Cronbach's alpha values of all variables are >0.8, indicating that the scale has high internal consistency and good reliability. The values of the factor loading meet the requirements of being between 0.5 and 0.95, and there is a significant difference at the level of *p* < 0.01 (28). The average variance extract (AVE) is used to measure convergent validity. Construct reliability (CR) is used to measure structural validity. As shown in Table 5 the AVE values of all variables are >0.6, the square root value of AVE is greater than the correlation coefficient of the ranks, and the CR values are >0.9, indicating that the measurement model has good validity (29).

**Table 5:** Measurement items, loadings, validity and reliability.

**Variable**	**Cronbach's alpha**	**AVE**	**CR**	**KMO**
BA	0.860	0.668	0.923	0.899
GM	0.864	0.697	0.920	0.882
GB	0.860	0.651	0.903	0.870
GR	0.903	0.684	0.928	0.905
MG	0.872	0.631	0.905	0.870
SS	0.878	0.676	0.912	0.870

**Table 6:** Descriptive statistics.

**Variable**	**M**	**SD**	**BA**	**BP**	**GR**	**MG**	**SS**	**GM**	**GB**
BA	2.071	1.087	0.817						
BP	2.398	1.337	−0.287^**^	0.840					
GR	1.977	0.918	0.057	−0.099^**^	0.827				
MG	1.615	0.527	0.057	−0.133^**^	0.249^**^	0.794			
SS	2.166	1.237	0.010	−0.116^**^	0.276^**^	0.304^**^	0.822		
GM	2.072	1.117	0.454^**^	−0.466^**^	0.217^**^	0.231^**^	0.217^**^	0.835	
GB	1.908	0.839	0.373^**^	−0.383^**^	0.286^**^	0.317^**^	0.198^**^	0.551^**^	0.807

*p < 0.05;

** *p* < 0.01.

#### Hypothesis test

**Figure 3:**
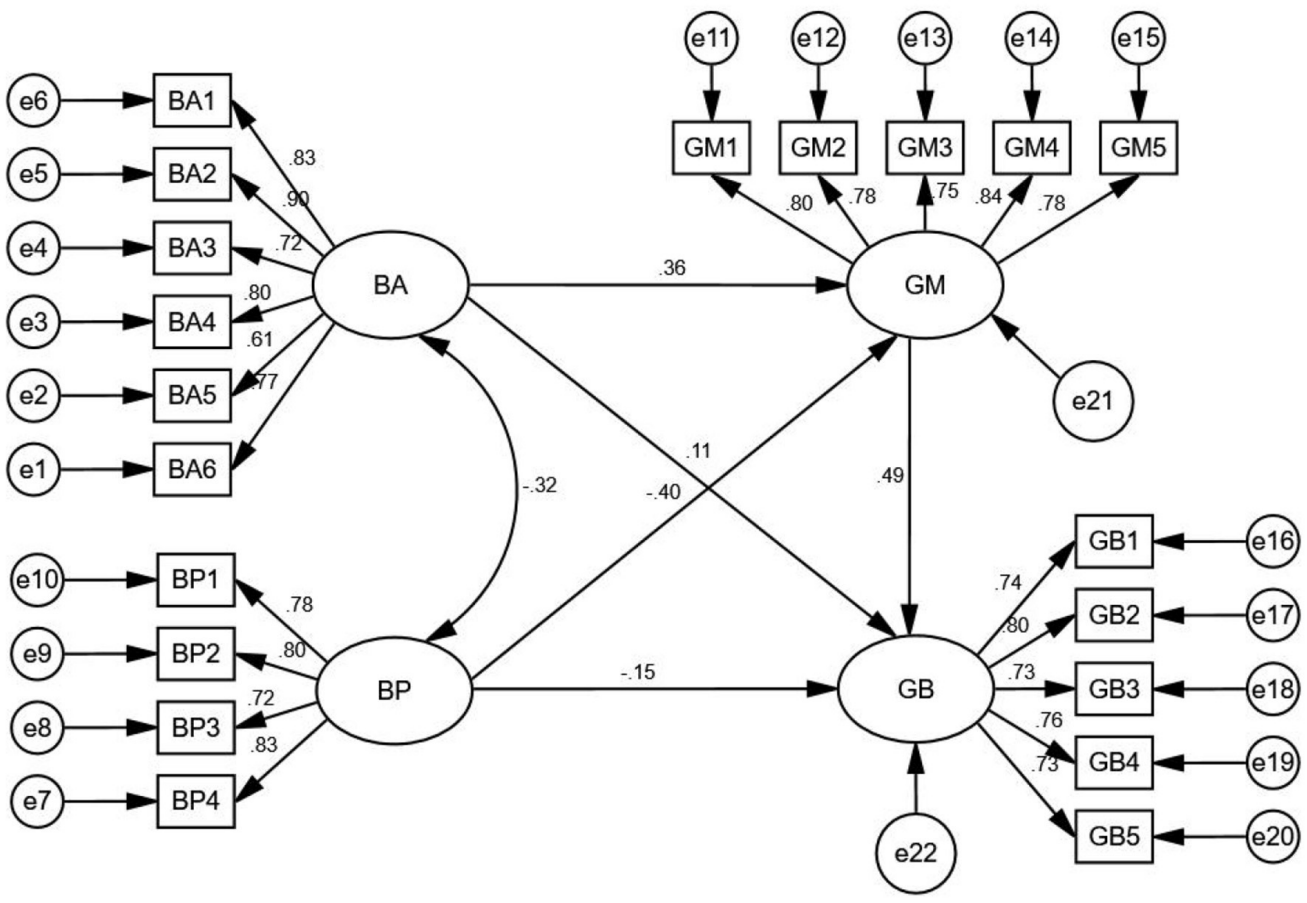
Model path diagram.

In this study, IBM SPSS AMOS 24 was used to analyze the direct and mediating effects (30). The structural equation modeling is shown in Figure 3. It can be seen from Table 7 that the measurement model is consistent with the survey data. Thus, the model shows a good fit to the data.

**Table 7:** Model suitability test table.

**Test statistics**	**Proximity value**	**Model test results**
RMSEA	<0.08	0.033
GFI	>0.90	0.959
NFI	>0.90	0.898
IFI	>0.90	0.984
AFI	>0.90	0.948
CFI	>0.90	0.959
PNFI	>0.50	0.775
PCFI	>0.50	0.803
Chi squared degree of freedom ratio	1 < CN < 3	2.842

The test results of the direct effect are shown in Table 8. Among the variables, five path coefficients are all significant (*p* < 0.01). Thus, H1, H2, H3, H4, and H5 are all established.

**Table 8:** Path inspection.

**Path**	**Non-standardized coefficient**	**Standardized coefficient**	**SE**	**CR**	***p*-value**
BA→ GM	0.245	0.363	0.028	8.862	***
BP→ GM	−0.249	−0.399	0.026	−9.423	***
BA→ GB	0.092	0.115	0.034	2.686	***
BP→ GB	−0.110	−0.148	0.034	−3.258	***
GM→ GB	0.586	0.490	0.064	9.134	***

***p < 0.001.

The mediating effects were tested based on the bootstrapping test method proposed by Ji et al. (8), and the test results are shown in Table 9. ① In the interaction relationship between BA and GB, with GM as an intermediary variable, the bias-corrected and percentile 95% confidence interval (CI) did not contain zero, *p* < 0.01, This result shows that the mediating effect of BA is significant, because BA has a significant direct effect on GB, and GM has a partial mediating effect in the interaction between BA and GB. ②In the interaction relationship between BP and GB, with GM as an intermediary variable, the bias-corrected and percentile 95% confidence interval (CI) did not contain zero, *p* < 0.01, This result shows that the mediating effect of BP is significant, because BP has a significant direct effect on GB, GM has a partial mediating effect in the interaction between BP and GB. Therefore, H6 and H7 are both established.

**Table 9:** Standardized bootstrap mediation effect test.

**Path**	**Effect**	**SE**	**Bias-corrected 95% CI**			**Percentile 95% CI**		
			**Lower**	**Upper**	***p*-value**	**Lower**	**Upper**	***p*-value**
BA-GM-GB	0.178	0.027	0.132	0.235	0.001	0.136	0.224	0.001
BP-GM-GB	−0.196	0.027	−0.253	−0.145	0.001	−0.242	−0.151	0.001

The Process plug-in of SPSS software was used, and model type 1 was selected. The non-parametric percentile method of bias correction was used for analysis. Bootstrapping consisted of 5,000 iterations, and the bias-corrected effect test results are shown in Tables 10–**12**.

**Table 10:** Government supervision and regulation test.

**Pathway**	**Green pesticide application behavior**		**Green pesticide application behavior**	
	**Beta**	***t-*value**	**Beta**	***t-*value**
GM	0.513	15.712	0.494	15.300
GR	0.174	5.343	0.161	5.001
GM × GR			0.156	4.902
R2	0.332		0.356	
F	163.566***		120.878***	

***p < 0.001.

Table 10 shows that the interaction terms between GR and GM is significant (β = 0.156, *p* < 0.001), indicating that GR plays an intermediary role between GM and GB. Table 11 shows that the interaction term between MG and GM is significant (β = 0.154, *p* < 0.001), indicating that MG plays an intermediary role between GM and GB. Table 12 shows that the interaction term between SS and GM is not significant (β = 0.019, *p* > 0.1), indicating that SS does not play a regulatory role between GM and GB.

**Table 11:** Market adjustment guidance test.

**Path**	**Green pesticide application behavior**		**Green pesticide application behavior**	
	**Beta**	***t*-value**	**Beta**	***t*-value**
GM	0.505	15.508	0.483	14.951
MR	0.201	6.172	0.181	5.623
GM × MR			0.154	4.831
R2	0.342		0.364	
F	170.414^***^		125.253^***^	

***p < 0.001.

**Table 12:** Test of social regulation effect.

**Path**	**Green pesticide application behavior**		**Green pesticide application behavior**	
	**Beta**	***t*-value**	**Beta**	***t*-value**
GM	0.533	16.052	0.53	15.664
SR	0.082	2.461	0.08	2.401
GM × SR			0.019	0.564
R2	0.31		0.31	
F	147.424^***^		98.286^***^	

***p < 0.001.

**Figure 4:**
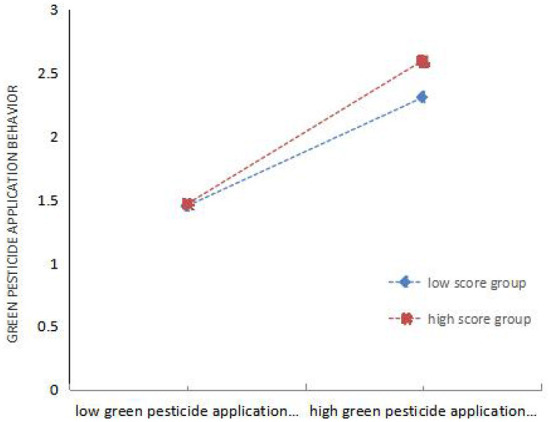
Regulatory effect test of government regulation and guidance.

**Figure 5:**
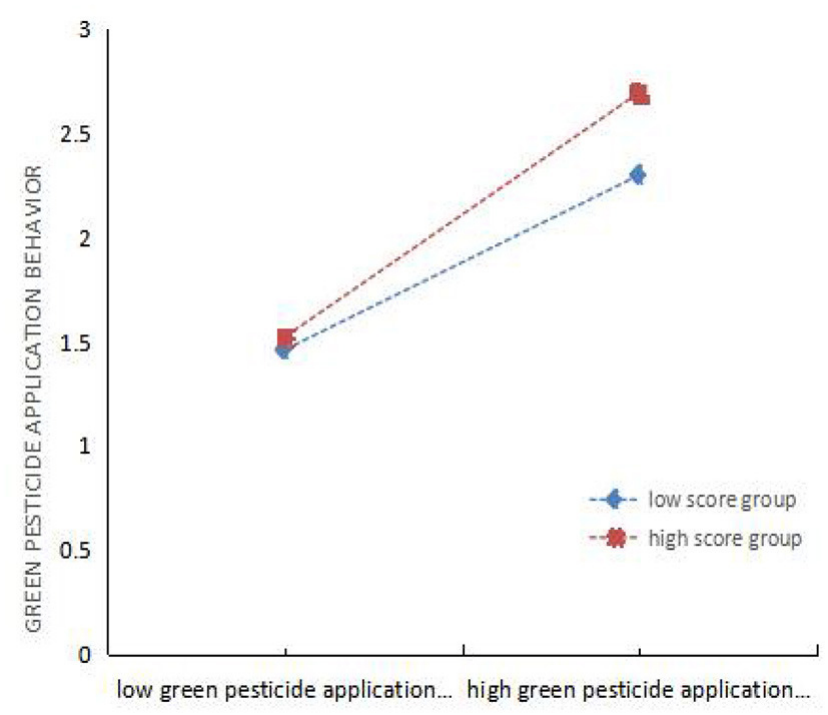
Regulatory effect test of Market adjustment guidance.

The purpose of drawing the mediating effect diagram is to intuitively show the intermediary role of GR and MG (see Figures 4, 5). Both GR and MG positively mediate the relationship between GM and GB. These conclusions are consistent with the data analysis results in Tables 10, 11. Therefore, H8 and H9 are valid, while H10 is not.

## Discussion

In-depth interviews were conducted to explore the influencing factors of vegetable farmers' green pesticide application behavior (GB), and the grounded theory was used to construct a conceptual model of the influencing factors.

Internal factors in behavior: behavioral attitude (BA), behavioral feedback perception (BP), and green pesticide application motivation (GM) are the internal factors of green pesticide application behavior (GB).

From the research results, vegetable farmers' pesticide residue awareness (PRA), pest and disease awareness (PDA), environmental pollution awareness (EPA), health hazard awareness (HHA), social responsibility awareness (SRA), and knowledge ability level (KAL) have a positive impact on their GM, and these factors further promote their GB. When vegetable farmers have sufficient pesticide residue awareness (PRA), vegetable farmers have a relatively strong GM. Although vegetable farmers have a certain understanding of pests and diseases, as well as the yield and appearance of vegetables, compared with the destruction of the natural ecological balance, they pay more attention to the former. This phenomenon is the result of the combination of vegetable farmers' rationality and irrationality. Vegetable farmers have better environmental pollution awareness (EPA), they believe that pesticide residues cause river, well and soil pollution, and that the improper disposal of pesticide wastes seriously threaten the environment. They have insufficient knowledge of excessive pesticide residues. As a result, vegetable farmers' GM is not strong enough. Vegetable farmers lack the social responsibility awareness (SRA) and the knowledge ability level (KAL) is limited, weakening the subjective initiative of vegetable farmers to apply green pesticides to a certain extent, and inhibiting their GB.

BP has a negative effect on vegetable farmers' GM, and thus inhibits their GB. The BP conforms to human information processing, it is vegetable farmers' perception of their previous personal experience and practical effect feedback, as well as their practice prediction, determining the generation of GM. According to the interview results, most vegetable farmers expressed their willingness to adopt GB, on the premise that their inner needs must be met. According to the commodity smallholder theory proposed by Huang Zongzhi, smallholders' decision-making behavior is affected by both economic and non-economic factors. The GB of vegetable farmers must consider the influence of their own economic interests; if green pesticide application behavior can reduce costs, increase income and prevent danger, then vegetable farmers will adopt GB. If the cost of GB is too high, the profits are too low or risks are incurred, rational vegetable farmers will not adopt GB. Only when vegetable farmers' GB feedback results meet their inner needs, will they actively choose GB, otherwise, their green pesticide application motivation will be greatly reduced or even be zero. At the same time, over the course of many years of farming, the application behavior of vegetable farmers has become habitual, meaning that it is difficult to change.

External factors in behavior: government supervision and regulation (GR), market adjustment guidance (MG) and social reference norms (SS) are the external factors of green pesticide application behavior (GB).

GR is the external factor in vegetable farmers' GB, and it positively mediates the relationship between GM and GB. In the interviews, most vegetable farmers expressed that they did not know the laws and regulations related to the quality and safety of agricultural products; therefore, they had little or no impact on them. However, most vegetable farmers said that they would adopt green pesticide application if China's legal and regulatory enforcement (LRE) was strict and established a quality traceability system (QTS) to hold them accountable for their non-green pesticide application behavior. If the output of green vegetables is lower than ordinary vegetables, government subsidies can make a difference by ensuring that vegetable farmers do not suffer losses and by even providing additional rewards, so that vegetable farmers will be willing to adopt green pesticides application. In the incentive subsidy support (ISS), it is necessary to combat corruption to ensure the effective implementation of support policies. In addition, due to the lack of pesticide operation standardization (POS) and the chaos in pesticide market orders, fake pesticides and highly toxic pesticides still exist. Additionally, vegetable farmers are limited by their ability, and as a result, they randomly purchase pesticides.

MG is an external factor in vegetable farmers' GB, and it positively mediates the relationship between GM and GB. Realizing a quality control entry market (QCEM), quality mark identification (QMI), price based on quality (PBQ) and key risk control (KRC) can effectively improve vegetable farmers' green pesticide application motivation (GM), so that they actively choose GB. Market supervision departments mainly collect stall fees and undertake the task of clearing stores in the fragmented market, and the quality and safety of vegetables are not inspected and supervised. Notably, farmers use advanced methods to obtain good sales channels, and some vegetable farmers use WeChat groups and circles of friends to sell vegetables. While attracting many customers, they can also sell at a good price, which stimulates vegetable farmers' enthusiasm for green pesticide application. The information asymmetry between vegetable farmers and consumers, and consumers' lack of knowledge about vegetable quality and safety, suggest that consumers like to buy vegetables that have a beautiful appearance or that taste good. However, they do not know that the beautiful appearance of vegetables may be due to enlargers, sweeteners, ripening agents and so on. Vegetables that do not use pesticides randomly are not beautiful and have an average taste. In addition, the price of high-quality and safe vegetables is relatively high, and as a result, consumers have insufficient green vegetable recognition, acceptance and purchasing power.

Although some of the hypotheses are not empirically verified, it is undeniable that social factors can enhance the relationship between GM and GB. At present, the interviews found that there are various media publicity methods for agricultural product quality and safety, but the effect is not good. Public supervision and reports have a deterrent effect on vegetable farmers, who are afraid of being punished. If media exposure is used, the concept of smallholders will cause ambivalence among vegetable farmers in regard to using pesticides: on the one hand, they will be afraid of being laughed at by neighbors because of their poor harvest; on the other hand, in most rural areas, people are almost always familiar with each other, exposure will make them become the object of comment. In rural society, the group convergence effect brought by conformity behavior and shortcut behaviors are suppression and promotion factors in vegetable farmers' GB. In particular, neighbors and large vegetable farmers play an indispensable role in the transformation and spread of vegetable application technology. Small-scale vegetable farmers often tend to envy and venerate large vegetable farmers. The successful planting experience of large farmers plays a leading role in demonstration, allowing small-scale vegetable farmers to quickly see the benefits. Social moral constraints (SMC) can restrict vegetable farmers' GB to a certain extent, and it is morally acceptable to spray pesticides on vegetables.

## Conclusions

### Research conclusion

Conducting open coding, spindle coding and theoretical coding of the records of in-depth interviews with vegetable farmers', we explore vegetable farmers' GB and construct a green pesticide application factor model. The qualitative phase of our research shows that the factors in vegetable farmers' GB can be summarized as internal and external factors. Among them, the internal factors include behavioral attitude (BA), behavioral feedback perception (BP), and green pesticide application motivation (GM); external factors include government supervision and regulation (GR), market adjustment guidance (MG), and social reference specification (SS). The quantitative phase of our research shows that vegetable farmers' BA, BP and GM are internal factors in vegetable farmers' GB. Vegetable farmers' BA and BP directly determine the generation and strength of their GM, and indirectly affect GB. As external factors, GR and MG play an intermediary role in the process of generating and enhancing GM and then selecting GB. Strict control by the government, and quality control by the market have an important impact on the vegetable farmers' green pesticide application motivation, thus affecting their green pesticide application behavior. However, social factors cannot be ignored, and it is necessary to improve the existing social environment to encourage vegetable farmers to use green pesticides.

### Driving strategy

To improve the quality and safety of agricultural products, not only are the complementary advantages of the government, the market and society necessary, but it is also necessary to fully mobilize the enthusiasm of consumers to jointly drive the evolution of vegetable farmers from non-green pesticide application behavior to GB. Starting from multiple perspectives and levels, we construct a multi-agent driving strategy for vegetable farmers to evolve from non-green pesticide application behavior to GB. On the basis of clarifying the influence mechanism of relevant factors on farmers' green pesticide application behavior, the multi-center governance theory, coordination theory and mechanism design theory are used for reference to build a multi governance mechanism for farmers' green pesticide application behavior. The main strategic implications are as follows:

First, government should improve the laws and regulations, strengthen the enforcement of laws and regulations, establish a reward and punishment mechanism, strengthen publicity and education, promote pesticide application standards, pesticide operation standardization, implement pesticide subsidies, establish vegetable quality and safety traceability system, popularize pest prevention technology, promote advanced pesticide, severely combat corruption, and other angles, use administrative measures to supervise and guide vegetable farmers' pesticide application behavior.

Second, give full use to market regulation and control. Focus on regulating the organized physical market, combating strictly the non-contracted non-physical market, improving the organization of the non-organized physical market, strictly inspecting the vegetables entering the market to control the vegetable quality before entering the market. Especially off-season vegetables planted in greenhouses are the key prevention and control targets, and reject unqualified vegetables out of the market. At the same time, introduce and improve the corresponding quality mark identification to distinguish vegetables into different grades. Promote high quality and high price, and encourage consumers to purchase qualified vegetables to reduce the worries of vegetable farmers.

Third, strive to strengthen the social reference effect and let it play its role. Media communication should adopt various modes such as emotional prayer, fearful prayer, relevance prayer, practical experiential activity appeal, moral prayer and others, so that make vegetable farmers empathize and form social moral constraints. Improve the reporting system, and make full use of modern information technology to facilitate the masses to report.

Fourth, avoid information asymmetry among multiple subjects and realize information sharing among multiple subjects. Government departments can establish a complete set of authoritative information publishing and sharing platforms, specific methods can take government departments jointly authorize an information service management agency, collect agricultural product quality and safety market information timely, summarize, identify and analyze timely. Establish and improve agricultural product quality and safety market information warning system and information network platform. Based on this, establishing a multi-agent driving strategy is the only way to promote the evolution of vegetable farmers from non-green to green pesticide application behavior.

## Data availability statement

The original contributions presented in the study are included in the article/supplementary material, further inquiries can be directed to the corresponding author.

## Author contributions

Conceptualization: YT. Methodology and writing—original draft preparation: YT and XC. Investigation: YT, XC, and YJ. Writing—review and editing: YJ, ZY, and XG. Supervision and project administration: YJ, ZY, and XG. Funding acquisition: ZY and YT. All authors have read and agreed to the published version of the manuscript.

## Funding

This work was supported by Humanities and Social Sciences Foundation of Ministry of Education of China (Grant 18YJC630162), Heilongjiang Province Postdoctoral Science Foundation (Grant LBH-Z17018), Key Laboratory Project of Modern Agricultural Equipment Technology in Northern Cold Region (Grant KF18-01), Young Talents of Northeast Agricultural University (Grant 20XG07), and Heilongjiang Philosophy and Social Sciences Research Planning Project (Grant 21JYD273).

## Conflict of interest

The authors declare that the research was conducted in the absence of any commercial or financial relationships that could be construed as a potential conflict of interest.

## Publisher's note

All claims expressed in this article are solely those of the authors and do not necessarily represent those of their affiliated organizations, or those of the publisher, the editors and the reviewers. Any product that may be evaluated in this article, or claim that may be made by its manufacturer, is not guaranteed or endorsed by the publisher.
